# Body-size Scaling is Related to Gut Microbial Diversity, Metabolism and Dietary Niche of Arboreal Folivorous Flying Squirrels

**DOI:** 10.1038/s41598-020-64801-y

**Published:** 2020-05-08

**Authors:** Po-Yu Liu, An-Chi Cheng, Shiao-Wei Huang, Hsiao-Pei Lu, Tatsuo Oshida, Wenhua Liu, Hon-Tsen Yu

**Affiliations:** 1 Genome and Systems Biology Degree Program, National Taiwan University and Academia Sinica, Taipei, Taiwan, Republic of China; 2 Department of Life Science, National Taiwan University, Taipei, Taiwan, Republic of China; 3 Laboratory of Wildlife Biology, Obihiro University of Agriculture and Veterinary Medicine, Obihiro, Japan; 4 Shaanxi Institute of Zoology, Xi’an, China; 5 Present Address: Department of Internal Medicine, National Taiwan University College of Medicine, Taipei, Taiwan, Republic of China; 6 Present Address: Department of Biotechnology and Bioindustry Sciences, National Cheng Kung University, Tainan, Taiwan, Republic of China

**Keywords:** Microbiome, Symbiosis, Animal physiology

## Abstract

Thermal homeostasis of mammals is constrained by body-size scaling. Consequently, small mammals require considerable energy to maintain a high mass-specific metabolic rate (MSMR) and sustain target body temperature. In association with gut microbiota, mammalian hosts acquire absorbable molecules and fulfill their metabolic requirements. Our objective was to characterize gut microbes in wild mammals and relate those findings to host body-size scaling. Two large (*Petaurista philippensis grandis* and *P. alborufus lena*), one medium (*Trogopterus xanthipes*) and one small (*Pteromys volans orii*) species of flying squirrels (FS) were studied. Using 16S rRNA genes, 1,104 OTUs were detected from four FS, with 1.99% of OTUs shared among all FS. Although all FS gut microbiota were dominated by *Firmicutes*, they were constituted by different bacterial families. Moreover, *Bacteroidetes* accounted for up to 19% of gut microbiota in small FS, but was absent in large FS. Finally, based on metagenome predictions, carbohydrate and amino acid metabolism genes were enriched in small body-size FS. In conclusion, gut microbiota compositions and predictive metabolic functions were characteristic of body-size in FS, consistent with their adaptations to folivorous dietary niches.

## Introduction

Body size is a major factor in endothermic animals’ metabolism to promote survival. Mammals metabolize foods to generate enough heat to balance surface heat loss^1^. However, heat losses depend on the body surface area to volume ratio; this constraint is called body-size (allometric) scaling and considered “structural and functional consequences of changes in size or scale among otherwise similar organisms”^2^. Basal metabolic rate (BMR) is the exponent of 3/4 or 2/3 of body mass (Kleiber’s law)^3,4^, whereas mass-specific metabolic rate (MSMR, in liters O_2_/kg/h) exponentially decreases as body mass increases (Fig. S1)^2,4,5^. Consequently, body size and energy metabolism fundamentally constrain the interaction between animals and their environment and determine their niche.

It remains unclear how small arboreal folivorous mammals maintain a high MSMR as they have small to moderate body sizes (~250 to 8200 g), due to both physical (habitat structures) and energetic (poor diet) limitations of living in treetops^6–8^. With a plant-based diet comprised of 50 to 85% fiber and no endogenous enzymes to digest plant biomass, small arboreal folivorous mammals must address their MSMR through adapted digestive strategies^9^. There are two apparent solutions: 1) increased retention time of digesta in enlarged digestive chambers (e.g. cecum); and 2) assisted digestion from symbiotic gut microbes^10^. Mammals have suites of gut microbes to improve energy uptake^11–13^, enabling hosts to acquire absorbable molecules. For example, short-chain fatty acids (SCFAs) produced by gut microbes provide their hosts (e.g. sheep and cattle) with up to 70% of their caloric requirements^14^.

Variation of gut microbial composition is associated with hosts’ physiological circumstances, especially diet^15,16^. Diverse gut-microbial profiles converge according to dietary types of mammalian hosts^13,17,18^. Hosts acquire fitness within specific dietary niches that are reflected in variation in several dominant microbial taxa, e.g. phyla *Firmicutes*, *Bacteroidetes*, *Actinobacteria* and *Proteobacteria*^13,17–19^. With these diversified symbionts, microbes colonizing mammalian gastrointestinal tracts may have adapted distinct functions. For example, gut microbiota of carnivores contain more genes for protein degradation, whereas those of herbivores have numerous genes for protein biosynthesis and plant fiber degradation^18^. Through cooperation with gut microbiota, herbivorous hosts acquire absorbable nutrient molecules. Microbes not only release extracellular enzymes to break down polysaccharides and proteins, but also ferment SCFAs to provide energy for hosts^20^. Although cellulolytic gut microbes have been characterized (particularly from domesticated mammals), little is known about gut microbial diversity regarding wild mammals’ body-size scaling.

Leaf-eating flying squirrels are among the smallest arboreal mammals (range, 24 to 1500 g) and sustain a high metabolic rate (estimated as 0.41 to 1.82 liters O_2_/kg/h) despite a low-quality diet (*i.e*. tree leaves)^21–23^. Occupying specialized dietary niches in treetops, folivorous flying squirrels rely on symbiotic microbes in an enlarged cecum to degrade celluloses to meet energy demands^20^. To address scaling issues, we studied four species (from three genera) of folivorous flying squirrels, with body mass representing three size classes: 1) large (two species), *Petaurista philippensis grandis* (PPG; ~1300 g) and *P. alborufus lena* (PAL; ~1500 g); 2) medium. *Trogopterus xanthipes* (TX; ~450 g); and 3) small, *Pteromys volans orii* (PVO; ~130 g)^24–27^. Mass-specific metabolic rates (MSMR) of mammals (measured by oxygen consumption) are well studied, i.e., the so-called mouse-elephant curve (Fig. S1). The curve is useful because we conducted field studies, making direct measurements for MSMR not feasible. For these species, MSMR were estimated as 0.42 (PPG), 0.40 (PAL), 0.59 (TX), and 0.90 (PVO) liters O_2_/kg/h, respectively (Fig. S1). The MSMR of small flying squirrels is more than double that of large species. Therefore, it was anticipated that small folivorous flying squirrels had very effective digestion-absorption strategies. Lignocellulose, which constitutes the majority of plant biomass, is the major dietary component of these folivorous flying squirrels. Large flying squirrels (PPG and PAL) consume primarily leaves of broadleaf trees, accounting for up to 74.0% of their annual diet^28,29^. Captive TX in this study were fed natural diets, including leaves of Chinese arborvitae (*Platycladus orientalis*), pine nuts, and acorns^30,31^, whereas wild PVO consumed young leaves, buds, flowers, and seeds of *Salix* spp. and *Picea* spp.^25^.

Our objectives were to investigate differences in gut microbiota composition among folivorous flying squirrels of various sizes. We hypothesized that gut microbiota and microbial energy metabolism are constrained by body-size scaling and that gut microbial composition and functions reflect dietary niches of each flying squirrel species. Consequently, we elucidated gut microbial composition of the four folivorous flying squirrels in reference to their body mass by sequencing bacterial 16 S rRNA gene libraries from fecal samples. Thereafter, we predicted the metagenome and pathways of energy metabolism contributed by gut microbiota.

## Materials and Methods

### Animals and fecal sample collection

Analysis of gut microbiota was done for four species of flying squirrels: Siberian Flying Squirrel (*Pteromys volans orii*, PVO; n = 19), Complex Toothed Flying Squirrel (*Trogopterus xanthipes*, TX; n = 4) and two species of Giant Flying Squirrels (*Petaurista philippensis grandis*, PPG; n = 3 and *P. alborufus lena*, PAL; n = 3). Fecal samples from PVO flying squirrels were collected during a mark-recapture study conducted from May to August in 2013 and 2014 at The University of Tokyo Hokkaido Forest in Furano City (Hokkaido, Japan). Permits for live trapping were approved by Hokkaido Government Kamikawa General Subprefecture Bureau (No. 45 in 2013 and No. 10 in 2014) and by The University of Tokyo Hokkaido Forest (No. A13-12 in 2013 and No. A14-07 in 2014). These samples were sequenced in our previous study^32^; sequence reads were retrieved from NCBI Sequence Read Archive database (accession numbers: SRX3793743- SRX3793761). Fecal samples of both PAL and PPG were collected from wild individuals in March 2013 and in March-April 2014 at Wulai District, New Taipei City, Taiwan (collection permissions No. 1023228856 and No. 1022101678 were granted by the Government of New Taipei City and Forestry Bureau, Council of Agriculture, Executive Yuan in 2013 and 2014), in accordance with the Wildlife Conservation Act^33^. The TX squirrels were farmed animals (Supplementary Text) and fed Chinese arborvitae (*Platycladus orientalis*) leaves, pine nuts, acorns, etc. (Shangluo, Shaanxi Province, China)^30,31^ For these animals, fecal samples were collected from their cages in May, 2012 and immediately preserved in RNAlater® (Thermo Fisher Scientific, Carlsbad, CA, USA) for subsequent bacterial genomic DNA extraction. We did not retain live animals for any sampling. Feces of TX squirrels are used in folk medicine (see Supplementary Text).

### DNA extraction, PCR, library preparation and sequencing

Bacterial genomic DNA was extracted from fecal samples (~200 mg) using QIAamp Fast DNA Stool Mini kit (QIAGEN, Valencia, CA, USA), following pathogen detection protocols. The V3-V4 region of the bacterial 16 S rRNA gene was PCR-amplified using barcoded forward primers (XXXXXXCCTACGGGNGGCWGCAG) and reverse primers (XXXXXXGACTACHVGGGTATCTAATCC); 6-bp barcodes were indicated by XXXXXX. The PCR was performed under the following conditions: 94 °C for 4 min, followed by 25 cycles of 94 °C for 30 s, 57 °C for 30 s and 72 °C for 30 s, with a final elongation step at 72 °C for 8 min. Amplified products of expected size (464 bp) were purified with QIAquick Gel Extraction Kit (QIAGEN) and DNA concentrations determined using Qubit^®^ 3.0 Fluorometer (Invitrogen, San Diego, CA, USA). Three amplicon libraries were constructed by pooling 20 samples with equal amounts of DNA for each library. The 16 S rRNA gene amplicons were pair-ended sequenced (2 ×300 bp) with 1 Gb qualified outputs per library using an Illumina MiSeq platform at Yourgene Bioscience Company (Taiwan).

### Sequence analysis

Raw data acquired from the three libraries were processed according to the Amplicon SOP v2 of the Microbiome Helper workflow (https://github.com/mlangill/microbiome_helper)^34^. Paired-end reads were trimmed of barcodes with Cutadapt 1.8.1^35^ (-g XXXXXX -G XXXXXX–discard-untrimmed; XXXXXX indicates 6-bp barcodes). Trimmed sequence data were processed with QIIME 2 v. 2019.4^36^. The ‘DADA2’ plugin embedded in QIIME2 were used to identify amplicon sequence variants (ASVs) from de-multiplexed sequence files (with parameters:–p-trunc-len-f 270–p-trunc-len-r 210–p-max-ee 3)^37^. Then, taxonomy was assigned by the ‘classify-sklearn’ function of ‘feature-classifier’ plugin with a Naïve Bayes Classifier trained on SILVA 132, using 99% OTUs full-length sequences of 16 S rRNA genes^38^. In total, there were 277,256 qualified sequences representing 3,455 taxonomic features across the 29 samples (range, 959 to 33,265 reads). Due to over-classification of ASVs and unclear taxonomy assignment for wild animals’ microbiota, we re-clustered representative ASV features in OTUs with 97% similarity using uclust v1.2.22q^39^ and re-assigned taxonomy with SILVA 123, using 97% OTUs full-length sequences of 16 S rRNA genes using blastn 2.6.0 + ^40^ with e-value 1e-5 and extracted best hit according to bitscore (using SILVA 123 database in order to further predict metagenome by Tax4Fun; see below). Finally, 1,104 OTUs were redefined by the alternative method. After fine-tuning for the ASV table, the 97% representative sequences were aligned with MAFFT^41^ using the ‘alignment’ plugin and variable positions were masked with ‘mask’ function. A phylogenetic tree was built with the ‘Fasttree’ function^42^ in the ‘phylogeny’ plugin and then rooted with the ‘midpoint-root’ function.

### Biodiversity and statistical analyses

Microbial community analyses were conducted with R package *vegan*^43^. A Kruskal-Wallis test in R software^44^, with α=0.05, was used for all statistical analyses and Dunn’s test for post-hoc comparisons. To normalize sequencing output among samples, we rarefied the ASV/OTU table to 959 reads per sample. Alpha diversity indices, Shannon index (H’) was calculated by ‘diversity’ function, species richness (S) was counted by ‘specnumber’ function and species evenness (J) was calculated by following formula $J=\frac{H{\prime} }{\mathrm{ln}\,S}$, Faith’s phylogenetic diversity then was calculated by ‘pd’ function of *picante* package. For beta diversity, dissimilarities among microbial communities were measured by Bray-Curtis distance and conducted with principal coordinates analysis (PCoA). PERMANOVA (permutational multivariate analysis of variance) with pairwise comparisons, ANOSIM (analysis of similarity)^45^ and ADONIS (permutational multivariate analysis of variance using distance matrices)^46^ were used to test heterogeneity of microbial communities among host species.

### Network analysis for identifying co-occurrence microbial community members

Network analysis of co-occurrence microbial community members was done with the R package *igraph*^47^. A co-occurrence matrix was constructed from the OTU table, according to SparCC correlation coefficients^48^ (≥0.3) between OTUs (calculated by ‘sparcc’ function of *SpiecEasi* R package^49^); these coefficients were also used for assessing length of edges on the network. The latter was conducted with the fast greedy modularity optimization algorithm^50^ to identify clusters in the network. Nodes with <5 connection degrees were removed from the network and hub nodes from each cluster were extracted for further community structure analyses.

### Metagenome and functional prediction

Tax4Fun^51^ was used to predict the metagenome, which was based on SILVA 123 16S database to evaluate potential functions of flying squirrels’ gut microbiota. An OTU table was used for predicting Kyoto Encyclopedia of Genes and Genomes (KEGG) Orthology (KO) relative abundances and being categorized by KEGG pathways. The FTU (fraction taxonomic units unexplained) scores were evaluated for reliability of metagenome prediction (0.21 ± 0.09; Fig. S2). Metabolic pathway enrichment analysis was conducted with the R package *gage*^52^ (testing by ‘gage’ function to test metabolic pathway enrichment by comparing equal gene abundance distribution for within FS categories and by comparing mean gene abundance among FS categories; significant pathways were identified from one-tailed tests for up-regulation). The P values from multiple-testing were adjusted with false discovery rate (FDR), with a p-value adjusting function embedded in the *gage* package; significances of enrichment analyses were defined by FDR q-value <0.05. Enrichment scores were calculated according to the gene-set enrichment analysis (GSEA) algorithm of DAVID bioinformatics resources^53,54^.

## Results

### Characterization of folivorous flying squirrel gut microbiota

The Illumina MiSeq platform generated a total of 3,665,256 high quality paired-end sequences, with an expected sequence length of 464 bp and an average of 9,561 non-chimeric reads/sample, ranging from 959 to 33,265 reads/sample. The SILVA NR123 SSU rRNA database was applied to identify a total of 1,104 OTUs by re-clustering ASVs to 97%-identity OTUs. On average (mean ± SD), PVO had the most bacterial OTUs (111.47 ± 106.07), followed by TX (108.50 ± 56.01), PPG (107.00 ± 33.87), and PAL (76.67 ± 30.89). Overall, 1.99% of OTUs were shared by all four species, whereas 2.72-55.1% of unique OTUs were present in each of the four species (Fig. S3A). Since the two species of *Petaurista* (PAL and PPG) shared a substantial proportion of OTUs (41.2%; 103 OTUs; Fig. S3B), a comparison was also done by combining the two *Petaurista*, resulting in 4.08% of OTUs shared in three genera and 12.3-55.1% unique OTUs present in each genus (Fig. S3C). The two species of large flying squirrels (*Petaurista*) were much more similar in gut microbiota than species of other genera, whereas PVO harbored more than four-fold unique OTUs compared to either of the other two genera.

**Figure 1:**
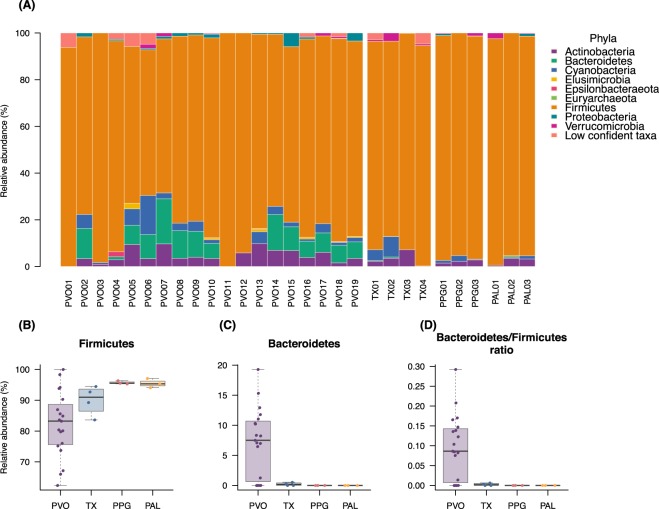
Relative abundance profiles of four folivorous flying squirrel species. (**A**) Phylum level relative abundance (%) barplots of gut microbiota from 29 FS individuals (19 PVO, 4 TX, 3 PPG, 3 PAL). Relative abundance of (**B**) *Firmiutes* and (**C**) *Bacteroidetes* grouped by four flying squirrel species. (**D**) *Bacteroidetes* to *Firmicutes* ratio of flying squirrel gut microbiota. PVO, *Pteromys volans orii*; TX, *Trogopterus xanthipes*; PPG, *Petaurista philippensis grandis*; PAL, *Petaurista alborufus lena*.

**Figure 2:**
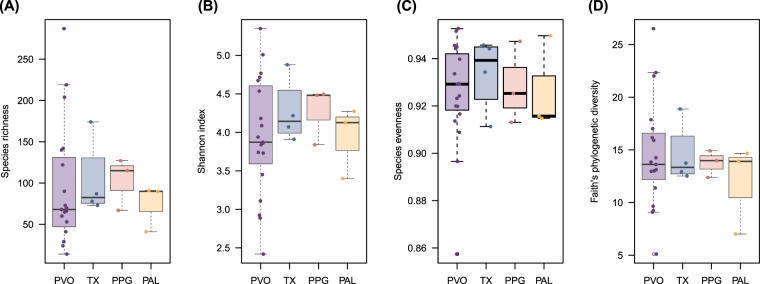
Alpha diversity indices of gut microbiota of four species of flying squirrels. (**A**) Margalef’s species richness index, (**B**) Shannon’s diversity index, (**C**) Pielou’s evenness index, and (**D**) Faith’s phylogenetic diversity. None of the indices differed among the four taxa, based on a Kruskal-Wallis test (p=0.69 for Margalef’s species richness index, p=0.76 for Shannon’s diversity index, p=0.95 for Peilou’s evenness index, and p=0.97 for Faith’s phylogenetic diversity). PVO, *Pteromys volans orii*; TX, *Trogopterus xanthipes*; PPG, *Petaurista philippensis grandis*; PAL, *Petaurista alborufus lena*.

Regarding relative abundance of all flying squirrel fecal microbiota (Fig. 1A), the most abundant microbial phylum identified was *Firmicutes* (average 62.36-100%), accounting for ~95% of gut microbiota in the two host species of *Petaurista* (Fig. 2, Fig. S1). The next dominant phyla were *Bacteroidetes* (0-7.2% on average), *Actinobacteria* (2.16-4.46% on average) and *Cyanobacteria* (0.63-3.42% on average; Fig. 1B,C and Fig. S4). Interestingly, *Bacteroidetes* was absent in the two species of *Petaurista*, but accounted for 0-19.29% in PVO and 0-0.52% in TX. It was noteworthy that from 1.21 (PVO) to 1.90% (TX) of OTUs were low confidently identified (<90% identity) in database and were suspected to be unknown prokaryotic species.

Additionally, a lower *Bacteroidetes* to *Firmicutes* ratio (B/F ratio) was regarded as obesity-related microbial biomarkers in laboratory models and human studies; in other words, the B/F ratio may reflect ability of energy extraction via gut microbiota^55,56^. The B/F ratios of four FS were measured (Fig. 1D). All FS had extreme low B/F ratio (less than 1 and close to 0), although PVO had a slightly higher B/F ratio (maximum 0.29). The FS B/F ratios are compared to other folivorous mammals (with higher B/F ratios) in the Discussion.

**Figure 3:**
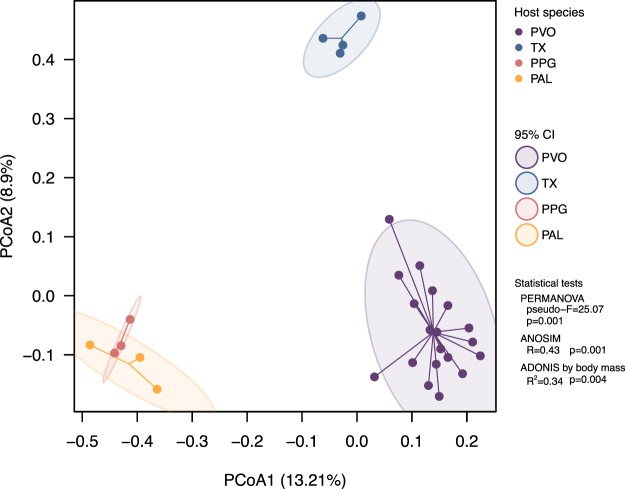
Principle coordinates analysis (PCoA) by Bray-Curtis distance metric of gut microbiota from four species of flying squirrels. Gut microbiota of flying squirrels are indicated by colors, *Pteromys volans orii* (PVO; purple), *Trogopterus xanthipes* (TX; blue), *Petaurista philippensis grandis* (PPG; red) and *Petaurista alborufus lena* (PAL; orange). Three distinct groups were distributed along the first two PCs. Shaded ellipses represented the 95% confidence intervals of FS groups. PERMANOVA, ANOSIM and ADONIS tests for the four host species differed (p=0.001, p=0.001, and p=0.004, respectively).

Although gut microbiota of PVO was more diverse in phylum composition, there were no differences (species level) among all host species in Shannon’s diversity index (*p*=0.76, Kruskal-Wallis test), Margalef’s species richness index (*p*=0.69), Pielou’s evenness index (*p*=0.95), or Faith’s phylogenetic diversity (*p*=0.97; Fig. 2). Further, we discussed host-specific order and lower levels of microbial composition by using co-occurrence network analysis (see below). A principal coordinates analysis (PCoA) (Fig. 3) presented gut microbial beta diversity among host species and had three clusters corresponding to three FS genera (PERMANOVA, pseudo-F=25.07, *p*=0.001).

### Network analysis identifies distinction among microbiota at lower taxonomic level

**Figure 4:**
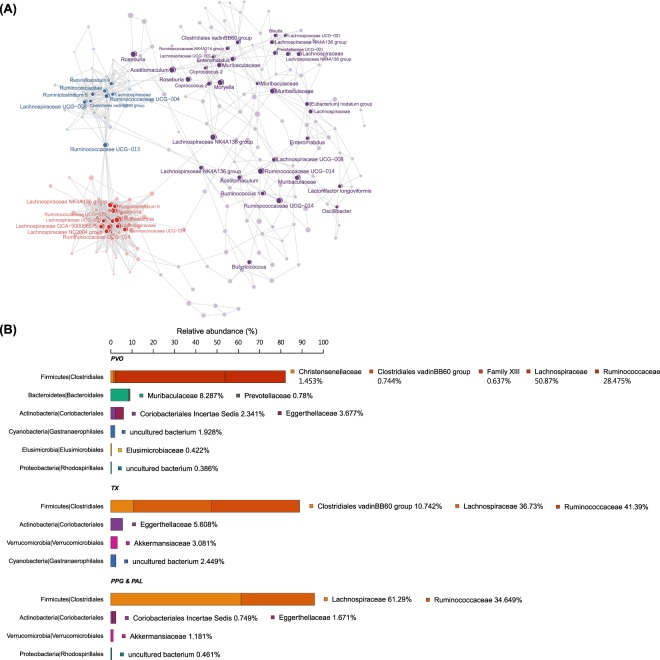
Core gut microbiota composition of three genera of flying squirrels. (**A**) Correlation network of OTUs in four species of flying squirrels. SparCC correlation (≥0.3) identified sub-communities based on a fast greedy modularity optimization algorithm. Nodes on the network were clustered into three groups delimited by host genus and body sizes; purple nodes: PVO/small FS featured OTUs; blue nodes: TX/medium FS featured OTUs; red node: PPG and PAL/large FS featured OTUs. Nodes with labeling species, genus, and/or family taxonomy names were hub/central OTUs within clusters which were identified by connection degree> the third quantile within a cluster. (**B**) Gut microbiota phyla/order/family stacked relative abundance barplots of three clusters (corresponding to PVO, TX and PPG & PAL) based on the correlation network. The phyla and orders are shown on the y-axis and bars filled with various colors represent families belonging to corresponding phylum and order.

Co-occurrence network analysis was done in addition to PCoA, because it not only identified clusters but also enabled visualization of taxa underlying distinctions among clusters (Fig. 4A). Clusters identified corresponded to the three genera in PCoA graph. Moreover, the network also identified centers (hubs) of core microbes at a finer taxonomic level. Taxonomy of the three clusters were profiled (Fig. 4B); all were dominated by families *Lachnospiraceae* and *Ruminococcaceae* which belong to order *Clostridiales* (>80%). Both PVO and TX clusters contained *Clostridiales* vadinBB60 group in their core microbiota (0.74 and 10.74%, respectively). However, only PVO cluster included additional featured families *Christensenellaceae* (1.43%), *Clostridiales* Family XIII (0.64%), *Elusimicrobiaceae* (*Elusimicrobia*, 0.42%), and two families under *Bacteroidales*, *Muribaculaceae* (was named as S24-7, 8.29%) and *Prevotellaceae* (0.78%). Family *Eggerthellaceae*, which belongs to (*Actinobacteria*, *Coriobacteriales*), was common in all FS and accounted for 3.68, 5.6 and 1.7% in small (PVO), medium (TX), and large (PPG and PAL) FS, respectively. Family *Coriobacteriales* Incertae Sedis which also belongs to phylum *Actinobacteria* and uncultured *Rhodospirillales* (*Proteobacteria*) bacteria were detected in both large (*Coriobacteriales*: 0.75%, *Rhodospirillales*: 0.46%) and small (*Coriobacteriales*: 2.34%, *Rhodospirillales*: 0.39%) FS. Conversely, *Akkermansiaceae* (*Verrucomicrobia*) was present in large (1.18%) and medium (3.08%) FS. *Gastranaerophilales*, which was named YS2, a non-photosynthetic *Cyanobacteria*, was present in small (1.39%) and medium (2.45%) FS.

We further identified hub OTUs of each cluster on the network according to node connectivity (third quantile or greater connection degrees within cluster; Fig. 4A). There were 33, 8, and 11 hub OTUs detected from PVO, TX, and PPG-PAL clusters, respectively. Although three clusters shared a large proportion of *Lachnospiraceae* and *Ruminococcaceae* at the family level, distinct genera and species served as key OTUs for discriminating microbial compositions of FS hosts with varying body sizes. For example, genera *Roseburia* and *Shuttleworthia* were two core microbes of the PPG-PAL cluster, as was *Ruminiclostridium* of the TX cluster, the genera *Roseburia*, *Acetitomaculum*, *Oscillibacter*, *Enterorhabdus*, *Moryella*, *Butyricicoccus*, *Coprococcus*, *Blautia*, *Acetitomaculum*, *Eubacterium nodatum* related genera, and *Lactonifactor longoviformis* of the PVO cluster.

### Folivorous flying squirrels’ gut microbiota harbored high energy producing potential and small flying squirrels’ microbiota enriched more diverse pathways

**Figure 5:**
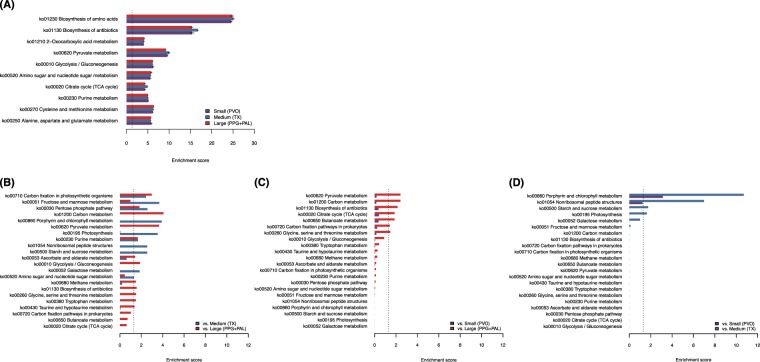
Enrichment analysis for predictive KEGG metabolic pathways of “within” and “between” flying squirrels’ gut microbiota. (**A**) Top 10 of “within” FS comparisons of KEGG metabolic pathways. All 43 significantly enriched pathways are shown in Fig. S5. (**B–D**) The “between” FS comparisons of KEGG metabolic pathways; (**B**) The enriched metabolic pathways of small FS by comparisons to medium and large FS, respectively; (**C**) medium FS by comparison to small and large FS; (**D**) large FS by comparison to small and medium FS. The dash lines indicate that the FDR q-value = 0.05. PVO, *Pteromys volans orii*; TX, *Trogopterus xanthipes*; PAL, *Petaurista alborufus lena*; PPG, *Petaurista philippensis grandis*.

Using Tax4Fun as a metagenome predictive exploratory tool, genes were categorized into KEGG Orthology metabolic pathways. All predicted KEGG Orthology (KOs) were mapped to 43 (“within” FS comparisons) and 94 (“between” FS comparisons) pathways among the 154 KEGG metabolic pathways. Each pathway was tested with gene-set enrichment by comparison to expected gene abundance within each FS category (“within” comparisons; Fig. 5A, Fig. S5, and Table S1) and mean gene abundance among FS categories (“between” comparisons; Fig. 5B–D and Table S1). There were 43 microbial metabolic pathways enriched in any or all of the three FS categories through the “within” comparisons). The top 10 enriched pathways were mainly related to carbohydrate and amino acid metabolism (Fig. 5A), followed by nucleotide, glycan, cofactor-vitamin and lipid metabolism pathways, and phytochemical or xenobiotic degradation (Fig. S5). For the “between” FS comparisons, various carbohydrate metabolic pathways in the small-FS gut microbiota were more enriched than in medium or large FS, such as fructose and mannose metabolism (ko00051), pentose phosphate pathway (ko00030), starch and sucrose metabolism (ko00500), glycolysis / gluconeogenesis (ko00010), and amino sugar and nucleotide sugar metabolism (ko00520); in addition, methane metabolism (ko00680) was also enriched in the small-FS gut microbiota (Fig. 5B). A few carbohydrate and amino acid metabolic pathways were more significantly enriched in medium- versus large-FS gut microbiota, e.g. pyruvate metabolism (ko00620), carbon metabolism (ko01200), TCA cycle (ko00020), butanoate metabolism (ko00650), prokaryotic carbon fixation (ko00720), and glycine, serine and threonine metabolism (ko00260) (Fig. 5C). However, only porphyrin and chlorophyll metabolism (ko00860) – a cofactor /vitamin metabolic pathway– was significantly enriched in the large-FS gut microbiota compared to both smaller flying squirrels’; and starch and sucrose metabolism was the only significant pathway of the large-FS gut microbiota that was enriched compared to medium FS.

## Discussion

### Strictly folivorous mammals may rely on gut microbiota to maintain a mass-specific metabolic rate

Folivorous mammals consume leaves with low nutrient content and have specific ecological niches and physiological adaptations^21,22^. Symbiotic gut microbiota likely have important roles to support ecological and evolutionary adaptations^16^. As small endothermic mammals have larger body surface area (BSA) to mass ratio, they consume energy to compensate heat loss at a faster rate than larger endothermic mammals in a resting condition. Furthermore, strictly folivorous arboreal mammals have a lower limit of body mass of ~1 kg^57^. Therefore, to maintain high MSMR, small leaf-eating mammals must rely either on a diet of high-nutrient content, or on a high digestion-absorption rate assisted by gut microbiota^4,57^. Our study contributed to understanding distinct gut microbiomes associated with folivorous FS of various body sizes, *i.e*., two large (PAL and PPG), one medium (TX), and one small FS (PVO), which reflect host’s diet niche and metabolic efficiency. Although distinction of FS gut microbial compositions may have been confounded by factors inherent to the hosts (e.g. taxonomy, physiology) or by environmental factors (e.g. diet, geographical distribution), this study was valid and valuable in terms of all sub-tribe Pteromyina flying squirrels (Sciuridae, Brandt, 1855) based on Kleiber’s law^3^ (i.e. body-size scaling).

### Flying squirrels with distinct body sizes and dietary niches had distinct gut microbiota

Nineteen (1.99%) gut microbial OTUs were shared among all four species of FS, whereas 54-103 (7.9-41.2%) OTUs were shared between any two FS species (Fig. S3). We re-analyzed data of Muegge *et al*. (2008) of six herbivorous hindgut fermenters housed in US zoos (two zebras, African elephant, two black rhinos, African wild ass, orangutan, and rabbit). There were 478 OTUs identified, but no OTUs shared among these divergent species. In contrast, four FS in this study are phylogenetic kin. Despite geographic disparity, they occupy similar ecological niches – inhabiting treetops and consuming leaf-based diets. The two large FS (PPG and PAL) with comparable body sizes harbored gut microbiota with similar composition (42.2%), whereas the medium-small FS in our study with discrete ranges of body sizes harbored distinct gut microbiota (Fig. 3). In the two large FS, the majority (95%) of gut microbiota were composed of the phylum *Firmicutes* with two dominant families, namely *Lachnospiraceae* (52.52%) and *Ruminococcaceae* (40.62%) (Fig. 4). Both of these families are common in mammalian guts, especially highly abundant in herbivores, due to their ability to degrade complex polysaccharides to SCFAs^58–60^. In contrast, in medium and small FS, *Firmicutes* (*Lachnospiraceae*, 46.27% and *Ruminococcaceae*, 32.79%) comprised only ~70% of gut microbiota. Based on a genomic comparison study, both of these two dominant bacterial families are common in gut environments and have similar fibrolytic functions^61^.

In addition, two minor *Firmicutes* (*Christensenellaceae*, 1.95% and *Clostridiales* vadinBB60, 2.78%) were also enriched in medium and small FS. It was reported that *Christensenellaceae* was significantly enriched in humans with a lean body mass index (BMI; < 25)^62^; however, *Clostridiales* vadinBB60 was enriched but *Christensenellaceae* was decreased in mice on a high-fat diet^63^. Based on our findings and previous studies, that *Clostridiales* vadinBB60 were enriched in medium-small FS implied that TX’s and PVO’s lipid-rich diet (pine nuts and seeds; see Introduction) in either captive or wild environments and that *Christensenellaceae* were more abundant in lean animals was consistent with greater heat losses in smaller body size FS.

There were up to 19.3% phylum *Bacteroidetes* in gut microbiomes of PVO. The *Bacteroidetes* family *Muribaculaceae* (was known as S24-7), accounting for ~8.3% of core microbiota in PVO, is a common bacterial family in herbivore gastrointestinal tracts; it has high potential for degradation of plant glycans and can be enriched by high-fat diets in laboratory mice^64–69^. Presumably, *Muribaculaceae* partially replaced the function of *Firmicutes* for cellulose degradation and may have promoted lipid absorption in gut microbiomes of TX and PVO that ate seeds with high lipid content^25,29–31,70^.

It is noteworthy that some bias may have occurred while assessing gut microbial composition in this study (also see Limitations and perspectives below). The four species of FS in this study are naturally distributed in disparate geographical regions of Asia (China, Japan, and Taiwan), and adapted to local environments, e.g. climate, phenology and different plant-source diet, which may also affect their gut microbial composition. Despite sampling bias and study design limitations, we tried to explore body-size issue of host-microbe interactions based on comparative physiology (Kleiber’s law). We limited research targets to four FS that shared largely evolutionary and ecological niches: shared common ancestors, adapted leaf-based diets and inhabited treetops.

### Bacteroidetes/Firmicutes ratio may be related to gut microbiota and host metabolism relationships

Regarding the *Bacteroidetes* to *Firmicutes* ratio of gut microbiota, increased *Firmicutes* was associated with obesity in a laboratory mouse model and humans^55,56,71^; however, wild animals, especially herbivorous mammals, harbor much more *Firmicutes* than *Bacteroidetes*^72–74^. For strictly leaf-based diet cases, folivorous mammals can be either foregut- or hindgut-fermenters, with distinct strategies to degrade a high-fiber diet^9^, thereby contributing to unique gut microbial communities. In the present study, as hindgut-fermenters, folivorous flying squirrels had a lower (0-0.29 in PVO; 0-19.3% of *Bacteroidetes* versus 62.4-100% of *Firmicutes*) or even zero (absence of *Bacteroidetes* in all large FS) ratio of *Bacteroidetes*/*Firmicutes* in their gut microbiota, in contrast to non-ruminant foregut-fermentative folivores (*e.g*. 23.2-88.4% of *Bacteroidetes* versus 10.2-51.79% of *Firmicutes* in Colobus monkey and langur; re-analyzed data from Ley *et al*. 2008)^13,75,76^. Other hindgut-fermentative folivores also had a low *Bacteroidetes*/*Firmicutes* ratio in their gut microbiota, *e.g*., 13.3% of *Bacteroidetes* versus 68.4% of *Firmicutes* in black Howler Monkey (*Alouatta pigra*)^77^. Taken together, we inferred that folivorous mammals independently acquired their own unique gut microbiota in response to distinct digestive strategies, *i.e*., foregut- or hindgut-fermentation^13^.

### Gut microbiota drive biomass conversions of leaf-based diets

Mammals have a variety of diets that create taxonomic and functional diversities of gut microbiota^13,18^. Variations in gut microbiota affect multiple aspects of host physiology^14^, especially adaptation for extracting energy from various types of feed^16^. Muegge *et al*. (2011) indicated that convergence of mammalian gut microbiota is related to dietary type instead of host phylogeny. However, there are few studies on variation in gut microbiota in relation to host body size and metabolic rate. In this study, we focused on gut microbiota of strictly folivorous, small mammals for two reasons: 1) leaf-based food sources, with a high fiber content, are expected to supply marginal nutrients; and 2) small mammals usually need to maintain a higher MSMR. Thus, we were also interested in gut-microbiota aided metabolic pathways of energy extraction from low-quality diets.

In previous studies, gut microbiota of two folivorous mammals (Yunnan Snub-Nosed Monkey *Rhinopithecus bieti*, foregut fermenter and *Petaurista alborufus lena*, hindgut fermenter) were enriched with carbohydrate metabolic pathway genes, the second most abundant orthologues after those involved in protein/amino acid metabolism^73,78^. With a high diversity of glycoside hydrolases (GHs), folivores can degrade a variety of lignocellulosic biomass from leaf-based diets. Valid microbes harbored in *Rhinopithecus bieti* were phyla *Bacteroidetes* (*Bacteroides vulgates*, and *B. fragilis*), *Fibrobacteres* (*Fibrobacter succinogenes*), and *Spirochaetes*, whereas *P. alborufus lena* had predominately *Firmicutes*^73,78^.

For this study, we predicted gut metagenomes of four species of folivorous flying squirrels. Irrespective of body size, carbohydrate and amino acid metabolism were potentially enriched in metabolic pathways. Gut microbiota harboring more energy-producing genes may have been due to high energy demand of small body-size mammals (*i.e*. PVO). The medium FS (TX), with three-fold higher estimated MSMR than large FS, had energy-producing enriched in microbiota, although the difference was not significant. Conversely, large FS microbiota were composed of>90% of *Firmicutes* bacteria (mainly from families *Lachnospiraceae* and *Ruminococcaceae*), which might harbor most energy-producing genes of whole large FS microbiome. Despite decreasing *Firmicutes* in medium and small FS microbiota, there were other sub-dominant phyla such as *Bacteroidetes* (Families *Bacteroidaceae*, *Prevotellaceae*, *Muribaculaceae*/S24-7), *Cyanobacteria* (Order *Gastranaerophilales*/YS2), *Proteobacteria*, and *Verrucomicrobia* (Family *Akkermansiaceae*) that may have complemented energy-producing functions or replaced *Firmitutes’* ecological roles in the gut.

In addition, as mentioned above, our metagenome prediction was consistent with previous studies that amino acid biosynthesis genes are the most abundant orthologues in herbivorous mammalian gut microbiota^18,73,78^. However, herbivorous diets may provide limited protein intakes and incomplete essential amino acid composition. Like most small hindgut fermenters, a digestive strategy perhaps used by folivorous flying squirrels is coprophagy (also known as cecotrophy). Many small mammals (most are rodents and rabbits) with plant-based, low-protein diets acquire nitrogen through coprophagy, which provides energy and increases protein uptake^9,79^. Nitrogen sources are mostly converted to amino acids by cecal microbes of small hindgut fermenters. Coprophagy by flying squirrels occurs in the wild (personal, unpublished observations). Moreover, based on metabolic function predictions, amino-acid-related genes were relatively complete in the gut microbiota of flying squirrels Therefore, we inferred that folivorous flying squirrels obtain essential nutrients (products of microbial metabolism) through coprophagy, similar to other small hindgut fermenters.

To cope with both poor diet and rapid heat-loses, arboreal folivorous flying squirrels adapted by nurturing suitable microbes in their enlarged ceca. Our findings provided insights regarding comparative physiology of thermal regulation would supported by adaptations of gut microbiota. We demonstrated that gut microbiota compositions were closely linked to differences in body sizes/MSMR in folivorous flying squirrels. In particular, microbial gene counts of metabolic pathways were also associated with body-size scaling of flying squirrels. Estimated high MSMR in small flying squirrels (PVO) would demand greater potential to extract energy by “co-operation” with gut microbiota. In addition, understanding adaptation of leaf-based dietary niche of flying squirrels may elucidate how microbial assistance enables these animals to function on low-quality diets. Large flying squirrels mutualizing with>90% cellulolytic microbes (*Firmicutes*) was consistent with their strictly leaf-based diet. In contrast, small flying squirrels harbored additional versatile phyla capable of cellulolytic activity as well as utilizing a high-lipid diet (*e.g. Eggerthellaceae*^80^). These results were consistent with the observation that a dietary adaptation of small flying squirrels is supplementing their leaf consumption with increased seed intake^29^. Further studies are required to characterize how dietary variations affect composition and function of gut microbiota.

### Limitations and perspectives

Although our study revealed interesting aspects of mammalian gut microbial symbionts regarding their diversities and metabolic potentials from the perspective of hosts’ body-weight scaling, results were preliminary and must be interpreted with precaution because our study remained largely exploratory in nature. First, more animals of each species involved could have been included and replicate species (at least two) of comparable sizes added, especially for medium and small body size, preferably from one locality with more uniform diets, either complete natural diet or one artificial diet. These measures would have prevented the compounding effects caused by geographical and diet heterogeneity which our current data cannot overcome. Second, some experimental approach must be drawn into the future study design, such as using species of small and large flying squirrels and applying some control of animals’ genetic backgrounds by using animals of the same or similar mitochondrial haplotypes (a commonly used genetic marker for studying wild animals). Finally, the resolution power of the prediction on metabolic pathway, although widely employed, is yet to be confirmed. It would be desirable to examine expression levels of a suite of enzymes that are relevant to energy production linked to gut microbial contributions.

## Supplementary information

Supplementary information.

## Data Availability

NCBI Sequence Read Archives: SRX3793597-SRX3793600, SRX3793722-SRX3793727, and SRX3793743- SRX3793761.
