# Supplementary material for: Body-size Scaling is Related to Gut Microbial Diversity, Metabolism and Dietary Niche of Arboreal Folivorous Flying Squirrels
Source: Sci Rep. 2020 May 8;10:7809. doi: 10.1038/s41598-020-64801-y (PMC7210948; doi:10.1038/s41598-020-64801-y)
Supplement: Supplementary file 1 — Supplementary information. [file 41598_2020_64801_MOESM1_ESM.pdf]

Body-size Scaling is Related to Gut Microbial Diversity, Metabolism and Dietary Niche of Arboreal Folivorous Flying Squirrels

Po-Yu Liu[1,2,$], An-Chi Cheng[2], Shiao-Wei Huang[2], Hsiao-Pei Lu[2,#], Tatsuo Oshida[3], Wenhua Liu[4], and Hon-Tsen Yu[1,2,*]

[1]Genome and Systems Biology Degree Program, National Taiwan University and Academia Sinica, Taiwan, Republic of China

[2]Department of Life Science, National Taiwan University, Taiwan, Republic of China

[3]Laboratory of Wildlife Biology, Obihiro University of Agriculture and Veterinary Medicine, Japan

[4]Shaanxi Institute of Zoology, Xi'an, China

$Present address: Department of Internal Medicine, National Taiwan University College of Medicine, Taipei, Taiwan, Republic of China

#Present address: Department of Biotechnology and Bioindustry Sciences, National Cheng Kung University, Tainan, Taiwan, Republic of China

[*]Correspondence:

Hon-Tsen Yu

Email: ayu@ntu.edu.tw; Tel: +886-2-33662453

Keywords: folivorous flying squirrel / mass-specific metabolic rate / gut microbiota / body-size scaling / cellulose degradation

Running head: Gut microbiota is related to allometry

Supplementary Text

The origin of farmed Complex Toothed Flying Squirrel (*Trogopterus xanthipes*)

In certain remote villages of the Chinese province of Shaanxi, flying squirrels are farmed on ethnobiological or herbological grounds. Feces of the species were recorded in the most notable Chinese medical literature "The *Compendium of Materia Medica*" (also known by the romanizations ***Bencao Gangmu***) written by Li Shizhen during the Ming dynasty; its first draft was completed in 1578. A convenient and brief reference is given by Wikipedia[1].

As one could imagine, the practice of farming has been going for years, if not for human generations, in villages throughout the region in Shaanxi. Farmers collect the feces to supplement their household income. This folk medicine has taken a new life and here we attached two articles that address the effective ingredients: one in Chinese with English abstract[2] and the other in English[3]. Today in China, flying squirrel's droppings are commercially available through the internet (Xi'an Tianrui Biotech Co., Ltd., Xi'an, Shaanxi Province, China), and it may be so in Korea, as Cho *et al.* (2013) may imply.

Feces of *Trogopterus xanthipes* (Wǔ Líng Zhī / Faeces Trogopteri / 五靈脂) was recorded as "bitter and sweet; disperse blood stasis, arrest pain and bleeding, and relieve toxin" in Chinese Materia Medica[4]. It means that the Faeces Trogopteri may promote blood circulation. The modern pharmacological studies demonstrated that its bioactive constituents involve many phytochemical derivatives, especially tricyclic diterpenes. One bioactive compound was named "wulingzhic acid" which was derived from conifers but isolated from Faeces Trogopteri. The compound is said to have properties of anticoagulation and antimicrobial[2].

Folk medicine such as using animal feces is an extremely cultural thing and could be very hard to apprehend across cultural backgrounds. One author (HT Yu) has spent time in the West (PhD from the University of California, Berkeley), and thus can appreciate the differences to attempt to bridge the cultural gaps. However, in recent years. this has become easier for outsiders to appreciate because increased interest and research in human microbiome. Fecal therapy is no longer far-fetched. We were led by our collaborator/coauthor, Wenhua Liu, who is a researcher of the Shaanxi Institute of Zoology to a remote village in the Province of Shaanxi to collect feces. Liu used to offer advice on animal husbandry for squirrels to villagers, who kindly permit us to collect feces from animals under their care. In any case, we did not retain any animals because they were properties of the villagers.

Interestingly, aborigines in Taiwan would consume flying squirrel droppings whenever possible (e.g., when hunting or they kept young squirrels collected from dens) and this habit has been passed down from the tribal elders to youngsters; yet they have not developed any farming practice like in Shaanxi or possibly in Korea.

References:

1 Compendium of Materia Medica. *Wikipedia* https://en.wikipedia.org/wiki/Compendium_of_Materia_Medica (2020).

2 Yang, D.-M., Su, S.-W., Li, X. & Zhu, T.-R. Studies on bioactive constituents from the excreta of Trogopterus xanthipes Milne-Edwards. *Acta Pharmaceutica Sinica* **22**, 756-760 (1987).

3 Cho, J. H. *et al.* 8beta-hydroxy-3-oxopimar-15-ene exerts anti-inflammatory effects by inhibiting ROS-mediated activation of the TRAF6-ASK1-p38 signaling pathway. *Immunopharmacol Immunotoxicol* **35**, 549-557, doi:10.3109/08923973.2013.820742 (2013).

4 Yang, J., Huang, H. & Zhu, L. J. *Introduction to Chinese Materia Medica*. (World Century, 2013).

Supplementary Figures

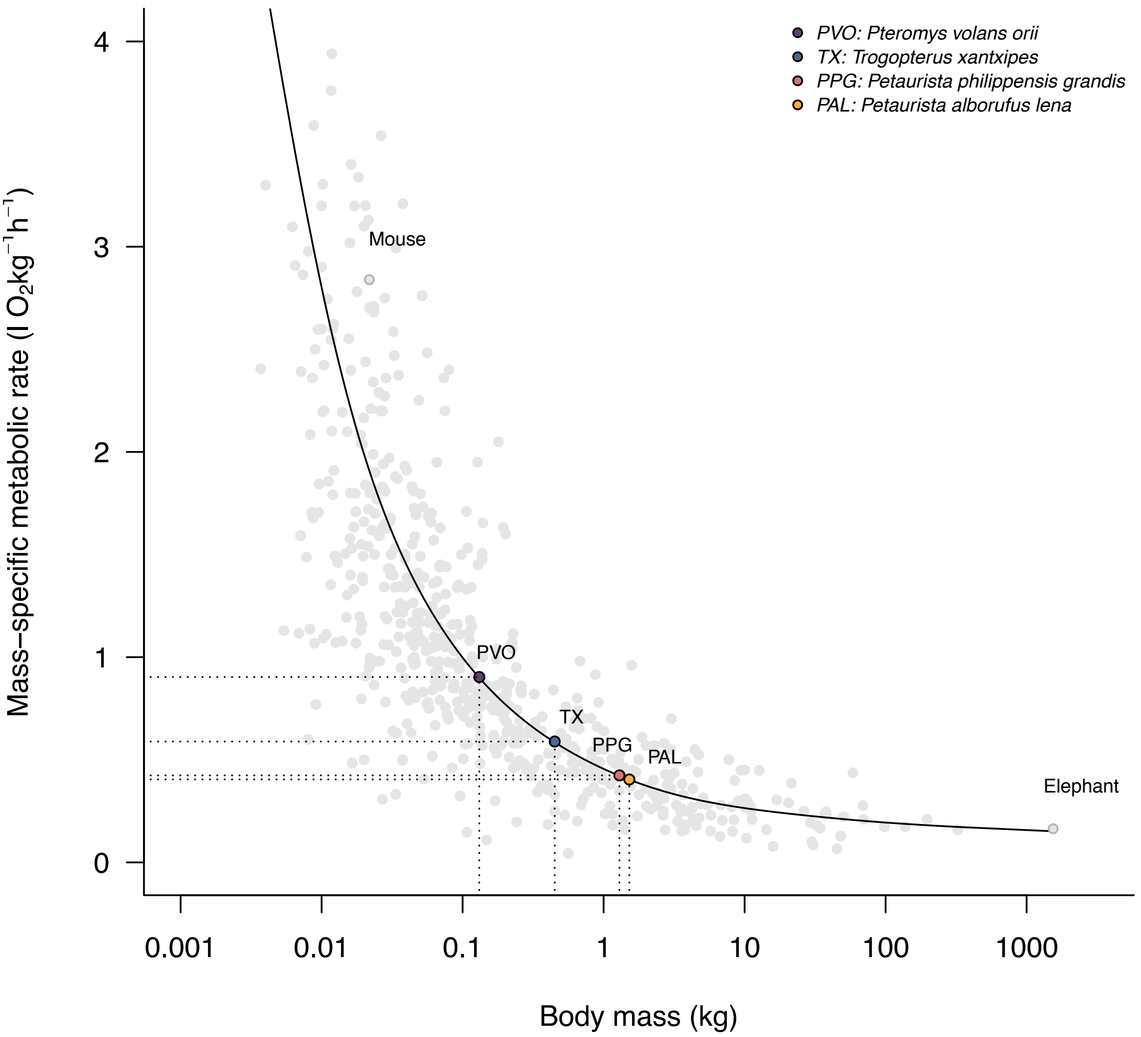

Figure S1. Specific oxygen consumption against body mass (mass-specific metabolic rate, MSMR) for 621 mammals and flying squirrels in this study – the "mouse-elephant curve," modified from Schmidt-Nielsen (1970). Mass-specific metabolic rates (MSMR) of mammals were measured by oxygen consumption (liters per kilogram per hour). The majority of data were retrieved from Krulwich & Bhatia (2015), with additional data retrieved from Kurta & Ferkin (1991), Langman *et al.* (1995) and White & Seymour (2003). The MSMR of four species of flying squirrels in this study were estimated based on average body mass.

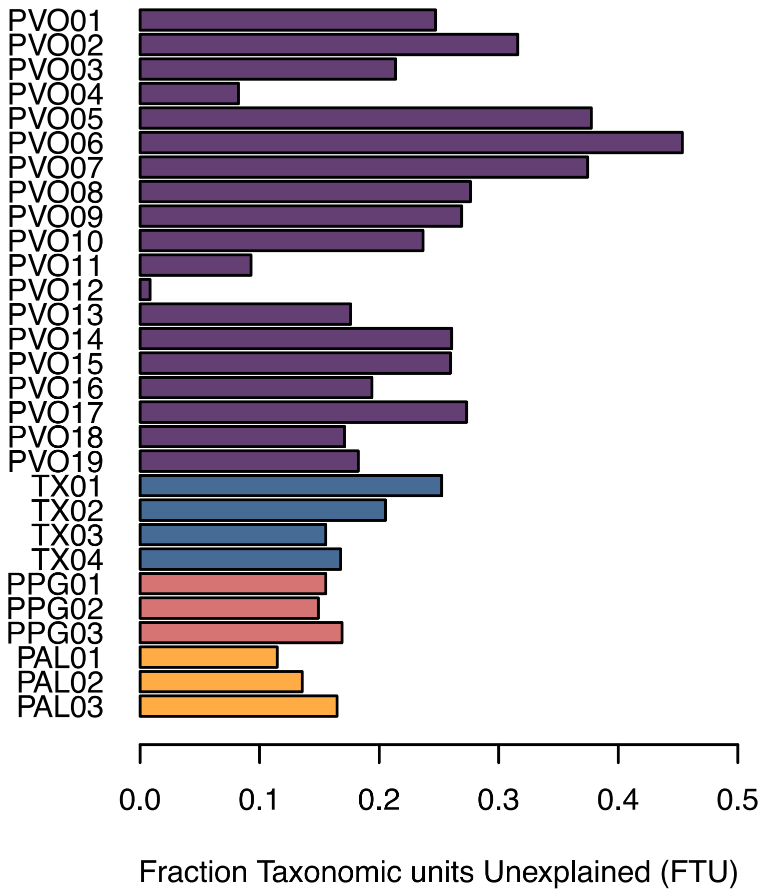

Figure S2. FTU scores of Tax4Fun metagenome prediction for 29 flying squirrel gut microbiota. The FTU (fraction taxonomic units unexplained), a score for evaluating metagenome prediction reliability (fraction of OTUs that could not be mapped to KEGG organisms). The FTU scores of 29 flying squirrels gut microbiota ranged from 0.008 to 0.45 (0.21±0.09).

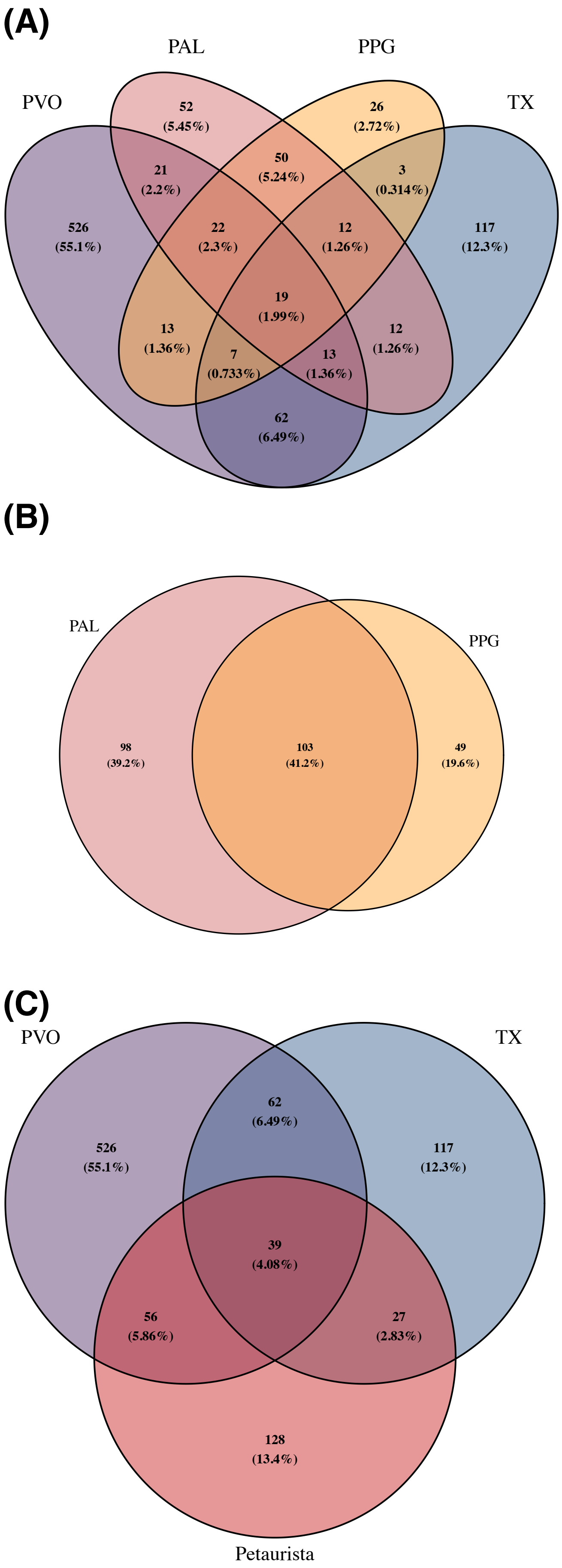
(A)
PAL
PPG
PVO
TX
52
(5.45%)
26
(2.72%)
50
(5.24%)
21
(2.2%)
3
(0.314%)
526
(55.1%)
22
(2.3%)
12
(1.26%)
117
(12.3%)
19
(1.99%)
13
(1.36%)
12
(1.26%)
7
(0.733%)
13
(1.36%)
62
(6.49%)
(B)
PAL
PPG
98
(39.2%)
103
(41.2%)
49
(19.6%)
(C)
PVO
TX
62
(6.49%)
526
(55.1%)
117
(12.3%)
39
(4.08%)
56
(5.86%)
27
(2.83%)
128
(13.4%)
Petaurista

Figure S3. Venn diagram of common and unique gut microbiota OTUs in four flying-squirrels. (A) There were 1,104 OTUs identified in the four species (PVO, *Pteromys volans orii*; TX, *Trogopterus xanthipes*; PAL, *Petaurista alborufus lena*; PPG, *Petaurista philippensis grandis*). There were 19 (1.99%) OTUs of common gut microbiota shared among all four species. Regarding gut microbiota unique to one species, PVO accounted for 526 (55.1%) OTUs, TX accounted for 117 (12.3%) OTUs, PAL accounted for 52 (5.45%) OTUs and PPG accounted for 26 (2.726%) OTUs, respectively. (B) The PAL and PPG shared 103 (41.2%) OTUs. Then OTUs from three PAL and PPG were combined in (C). (C) Proportions of both common and unique gut microbiota OTUs were increased after combination, with 4.08% OTUs shared for three genera and 13.4% OTUs unique for PAL & PPG.

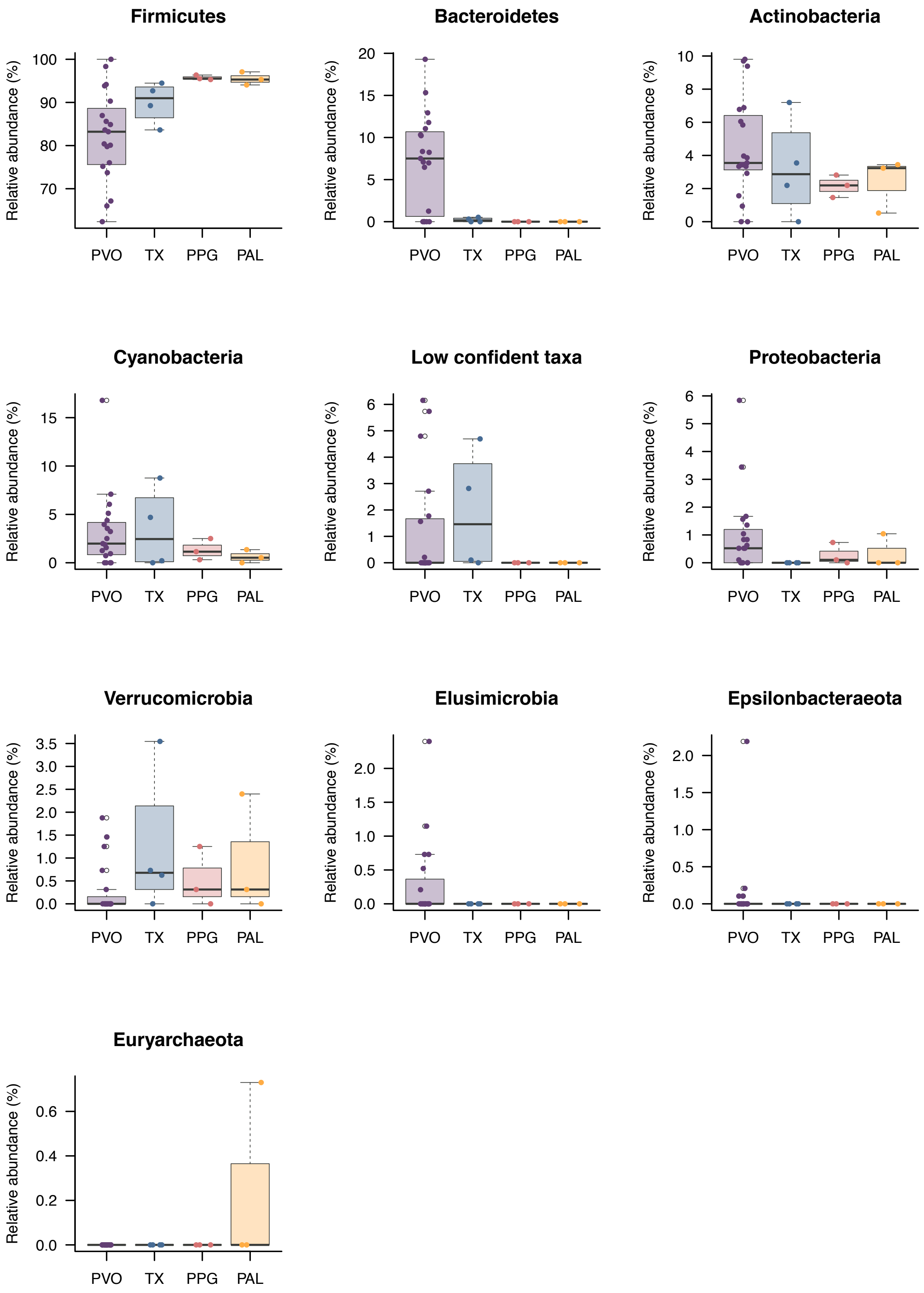

Figure S4. Boxplots of median relative abundance (%) of bacterial phyla in gut microbiota from four species of flying squirrels. There were nine major phyla of gut microbiota in the four host species, with *Firmicutes*, *Bacteroidetes*, *Actinobacteria*,

Cyanobacteria, *Proteobacteria*, and *Verrucomicrobia* being most abundant. Notably, *Bacteroidetes* were present only in PVO and TX, but absent from the two species of *Petaurista*. In contrast, two species of *Petaurista* harbored more *Firmicutes* than PVO and TX, whereas *Elusimicrobia* was presented only in PVO and Euryarchaeota was present only in PAL. PVO, *Pteromys volans orii*; TX, *Trogopterus xanthipes*; PAL, *Petaurista alborufus lena*; PPG, *Petaurista philippensis grandis*.

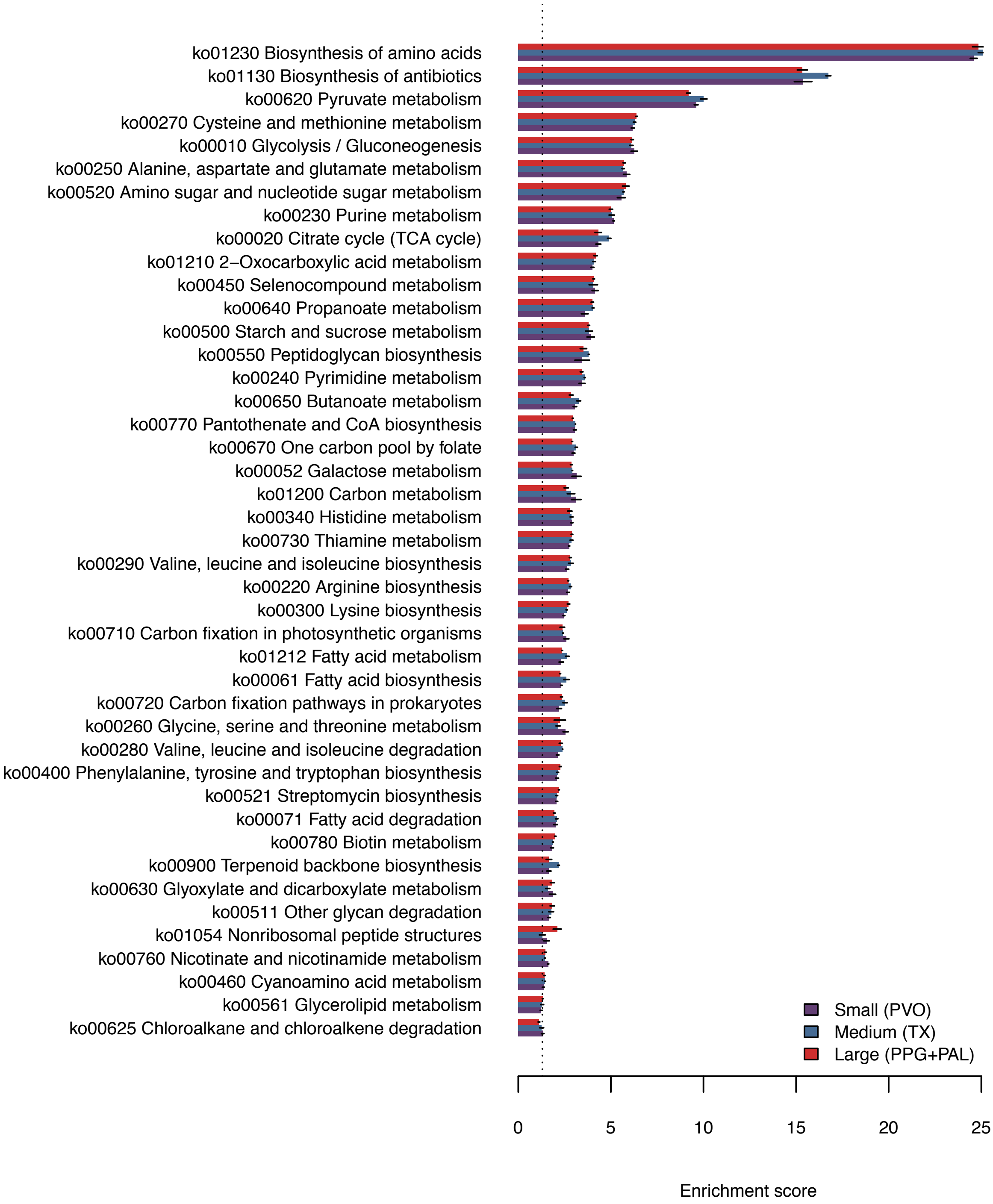

Figure 5. Enrichment analysis for predictive KEGG metabolic pathways "within" flying squirrels' gut microbiota. Forty-three KEGG metabolic pathways were enriched in the gut microbiota of any one of three body-size categories. The dash line indicates that the FDR q-value = 0.05. PVO, *Pteromys volans orii*; TX, *Trogopterus xanthipes*; PAL, *Petaurista alborufus lena*; PPG, *Petaurista philippensis grandis*.

Table S1. Predictive KEGG metabolic pathways abundance and enrichment scores of three sizes of flying squirrels.

There were 143 (“within” FS comparisons) and 94 (“between” FS comparisons) pathways identified from 154 level-3 KEGG metabolic pathways by enrichment analysis using ‘gage’ package in R software. PVO, *Pteromys volans orii* ; TX, *Trogopterus xanthipes* ; PAL, *Petaurista alborufus lena* ; PPG, *Petaurista philippensis grandis* .

KEGG pathway		Predicted abundance of metabolic pathways			Enrichment score within FS categories			Enrichment score between FS categories					
		Small FS	Medium FS	Large FS				Small FS vs.		Medium FS vs.		Large FS vs.	
Level 2	Level 3	(PVO)	(TX)	(PAL+PPG)	Small FS	Medium FS	Large FS	Medium	Large	Small	Large	Small	Medium
	ko01100 Metabolic pathways	1.60E-01	1.59E-01	1.60E-01	-	-	-	-	-	-	-	-	-
	ko01110 Biosynthesis of secondary metabolites	6.72E-02	6.76E-02	6.74E-02	-	-	-	-	-	-	-	-	-
	ko01120 Microbial metabolism in diverse environments	4.91E-02	4.90E-02	4.83E-02	-	-	-	-	-	-	-	-	-
	ko01130 Biosynthesis of antibiotics	5.26E-02	5.31E-02	5.23E-02	15.38±0.47	16.74±0.13	15.34±0.27	<0.01	1.56	0.37	2.14	<0.01	<0.01
**1.0 Global and overview maps**	ko01200 Carbon metabolism	2.63E-02	2.66E-02	2.56E-02	3.13±0.26	2.85±0.2	2.59±0.11	<0.01	4.08	0.17	2.42	<0.01	<0.01
	ko01210 2-Oxocarboxylic acid metabolism	7.70E-03	7.98E-03	7.93E-03	4.01±0.08	4.1±0.07	4.18±0.09	<0.01	<0.01	0.41	0.39	0.05	<0.01
	ko01212 Fatty acid metabolism	5.44E-03	5.63E-03	5.49E-03	2.32±0.12	2.65±0.1	2.37±0.02	<0.01	0.19	0.17	0.4	<0.01	<0.01
	ko01220 Degradation of aromatic compounds	2.50E-03	2.43E-03	2.44E-03	-	-	-	0.28	0.43	0.04	0.12	<0.01	<0.01
	ko01230 Biosynthesis of amino acids	3.48E-02	3.55E-02	3.53E-02	24.59±0.19	25.1±0.27	24.84±0.31	<0.01	<0.01	0.79	1.17	<0.01	<0.01
	ko00010 Glycolysis / Gluconeogenesis	1.27E-02	1.28E-02	1.25E-02	6.26±0.17	6.11±0.09	6.15±0.06	<0.01	1.88	0.05	0.89	<0.01	<0.01
	ko00020 Citrate cycle (TCA cycle)	7.25E-03	7.73E-03	7.10E-03	4.33±0.13	4.91±0.09	4.32±0.17	<0.01	0.63	0.41	1.86	<0.01	<0.01
	ko00030 Pentose phosphate pathway	5.68E-03	5.38E-03	5.47E-03	-	-	-	2.57	1.83	<0.01	0.1	<0.01	<0.01
	ko00040 Pentose and glucuronate interconversions	3.02E-03	3.03E-03	3.21E-03	-	-	-	<0.01	<0.01	0.05	0.02	0.03	0.03
	ko00051 Fructose and mannose metabolism	5.39E-03	4.99E-03	5.18E-03	-	-	-	3.67	0.97	<0.01	0.02	<0.01	0.11
	ko00052 Galactose metabolism	7.61E-03	7.27E-03	7.80E-03	3.15±0.25	2.92±0.01	2.87±0.05	1.85	<0.01	<0.01	<0.01	<0.01	1
	ko00053 Ascorbate and aldarate metabolism	9.06E-04	8.18E-04	7.88E-04	-	-	-	0.63	1.42	0.04	0.14	<0.01	<0.01
**1.1 Carbohydrate metabolism**	ko00500 Starch and sucrose metabolism	1.24E-02	1.19E-02	1.26E-02	3.91±0.2	3.82±0.19	3.81±0.05	2.55	<0.01	<0.01	<0.01	<0.01	1.76
	ko00520 Amino sugar and nucleotide sugar metabolism	1.18E-02	1.15E-02	1.17E-02	5.57±0.21	5.68±0.04	5.8±0.18	1.32	0.45	0.01	0.05	<0.01	<0.01
	ko00562 Inositol phosphate metabolism	6.15E-04	5.48E-04	6.03E-04	-	-	-	1.09	0.26	0.02	0.04	<0.01	<0.01
	ko00620 Pyruvate metabolism	1.43E-02	1.47E-02	1.39E-02	9.61±0.1	10.01±0.18	9.19±0.1	<0.01	3.67	0.18	2.42	<0.01	<0.01
	ko00630 Glyoxylate and dicarboxylate metabolism	8.28E-03	8.29E-03	8.28E-03	1.85±0.17	1.59±0.11	1.84±0.11	<0.01	0.27	0.05	0.12	<0.01	<0.01
	ko00640 Propanoate metabolism	7.34E-03	7.67E-03	7.31E-03	3.59±0.18	4.04±0.05	4±0.07	<0.01	0.46	0.37	1.24	<0.01	<0.01
	ko00650 Butanoate metabolism	9.43E-03	9.93E-03	9.32E-03	3.06±0.09	3.27±0.1	2.86±0.1	<0.01	0.71	0.37	1.86	<0.01	<0.01
	ko00660 C5-Branched dibasic acid metabolism	1.62E-03	1.67E-03	1.67E-03	-	-	-	<0.01	<0.01	0.06	0.12	<0.01	<0.01
	ko00190 Oxidative phosphorylation	6.68E-03	6.47E-03	6.50E-03	-	-	-	0.63	0.55	0.04	0.11	<0.01	<0.01
	ko00195 Photosynthesis	2.01E-03	1.83E-03	2.02E-03	-	-	-	3.53	0.04	<0.01	<0.01	<0.01	1.64
	ko00196 Photosynthesis - antenna proteins	8.16E-11	2.73E-10	0.00E+00	-	-	-	-	-	-	-	-	-
**1.2 Energy metabolism**	ko00680 Methane metabolism	8.04E-03	7.89E-03	7.73E-03	-	-	-	0.21	1.49	0.04	0.25	<0.01	<0.01
	ko00710 Carbon fixation in photosynthetic organisms	4.28E-03	4.06E-03	4.06E-03	2.6±0.14	2.41±0.02	2.38±0.12	2.44	2.98	<0.01	0.12	<0.01	<0.01
	ko00720 Carbon fixation pathways in prokaryotes	1.00E-02	1.04E-02	9.80E-03	2.2±0.13	2.53±0.12	2.33±0.06	<0.01	1.03	0.24	1.41	<0.01	<0.01
	ko00910 Nitrogen metabolism	5.43E-03	5.43E-03	5.57E-03	-	-	-	<0.01	<0.01	0.05	0.07	<0.01	<0.01
	ko00920 Sulfur metabolism	3.87E-03	3.71E-03	3.68E-03	-	-	-	0.63	0.91	0.04	0.12	<0.01	<0.01
	ko00061 Fatty acid biosynthesis	3.96E-03	4.01E-03	3.93E-03	2.33±0.04	2.6±0.15	2.26±0.02	<0.01	0.45	0.05	0.35	<0.01	<0.01
	ko00062 Fatty acid elongation	9.57E-08	1.16E-07	5.20E-08	-	-	-	-	-	-	-	-	-
	ko00071 Fatty acid degradation	4.56E-03	4.57E-03	4.56E-03	2.01±0.1	2.08±0.07	1.94±0.05	0.03	0.27	0.05	0.12	<0.01	<0.01
	ko00072 Synthesis and degradation of ketone bodies	6.34E-04	6.71E-04	6.44E-04	-	-	-	-	-	-	-	-	-
	ko00100 Steroid biosynthesis	5.07E-06	4.67E-06	2.71E-06	-	-	-	-	-	-	-	-	-
	ko00120 Primary bile acid biosynthesis	1.74E-04	1.56E-04	1.92E-04	-	-	-	-	-	-	-	-	-
	ko00121 Secondary bile acid biosynthesis	1.89E-04	1.66E-04	2.07E-04	-	-	-	-	-	-	-	-	-
	ko00140 Steroid hormone biosynthesis	7.14E-05	1.11E-04	7.32E-05	-	-	-	-	-	-	-	-	-
**1.3 Lipid metabolism**	ko00561 Glycerolipid metabolism	2.93E-03	2.86E-03	2.95E-03	1.24±0.06	1.29±0.08	1.32±0.02	0.44	0.12	0.04	0.05	<0.01	<0.01
	ko00564 Glycerophospholipid metabolism	2.92E-03	2.80E-03	2.98E-03	-	-	-	0.63	<0.01	0.04	0.02	<0.01	0.11
	ko00565 Ether lipid metabolism	4.64E-05	4.42E-05	5.44E-05	-	-	-	-	-	-	-	-	-
	ko00590 Arachidonic acid metabolism	9.60E-05	9.48E-05	9.45E-05	-	-	-	-	-	-	-	-	-
	ko00591 Linoleic acid metabolism	4.26E-07	8.61E-07	3.32E-08	-	-	-	-	-	-	-	-	-
	ko00592 alpha-Linolenic acid metabolism	1.57E-04	1.47E-04	1.60E-04	-	-	-	-	-	-	-	-	-
	ko00600 Sphingolipid metabolism	1.89E-03	1.96E-03	2.13E-03	-	-	-	<0.01	<0.01	0.06	0.08	0.05	<0.01
	ko01040 Biosynthesis of unsaturated fatty acids	8.80E-06	8.54E-06	5.26E-06	-	-	-	-	-	-	-	-	-
**1.4 Nucleotide metabolism**	ko00230 Purine metabolism	1.35E-02	1.32E-02	1.33E-02	5.15±0.04	5.05±0.14	5±0.09	1.69	1.67	<0.01	0.12	<0.01	<0.01
	ko00240 Pyrimidine metabolism	8.59E-03	8.53E-03	8.46E-03	3.44±0.16	3.59±0.03	3.42±0.07	<0.01	0.93	0.05	0.3	<0.01	<0.01
	ko00220 Arginine biosynthesis	5.75E-03	5.98E-03	5.78E-03	2.69±0.07	2.82±0.06	2.7±0.02	<0.01	0.21	0.38	1.11	<0.01	<0.01

1.5 Amino acid metabolism	ko00250 Alanine, aspartate and glutamate metabolism	1.04E-02	1.04E-02	1.04E-02	5.85±0.17	5.66±0.06	5.72±0.07	<0.01	0.12	0.05	0.16	<0.01	<0.01
	ko00260 Glycine, serine and threonine metabolism	7.47E-03	7.68E-03	7.32E-03	2.56±0.14	2.15±0.11	2.24±0.3	<0.01	1.51	0.17	1.46	<0.01	<0.01
	ko00270 Cysteine and methionine metabolism	9.96E-03	1.03E-02	1.01E-02	6.18±0.09	6.28±0.07	6.39±0.04	<0.01	0.04	0.41	1.05	<0.01	<0.01
	ko00280 Valine, leucine and isoleucine degradation	3.86E-03	4.10E-03	3.85E-03	2.13±0.07	2.4±0.01	2.3±0.08	<0.01	0.45	0.18	0.89	<0.01	<0.01
	ko00290 Valine, leucine and isoleucine biosynthesis	3.53E-03	3.66E-03	3.61E-03	2.64±0.09	2.84±0.13	2.81±0.05	<0.01	<0.01	0.37	0.4	<0.01	<0.01
	ko00300 Lysine biosynthesis	5.10E-03	5.13E-03	5.07E-03	2.47±0.05	2.62±0.04	2.72±0.07	<0.01	0.35	0.05	0.34	<0.01	<0.01
	ko00310 Lysine degradation	2.82E-03	2.91E-03	2.83E-03	-	-	-	<0.01	0.27	0.06	0.3	<0.01	<0.01
	ko00330 Arginine and proline metabolism	3.88E-03	3.93E-03	3.84E-03	-	-	-	<0.01	0.45	0.05	0.25	<0.01	<0.01
	ko00340 Histidine metabolism	3.17E-03	3.21E-03	3.18E-03	2.9±0.06	2.88±0.08	2.78±0.12	<0.01	0.21	0.05	0.2	<0.01	<0.01
	ko00350 Tyrosine metabolism	3.61E-03	3.62E-03	3.60E-03	-	-	-	<0.01	0.31	0.05	0.16	<0.01	<0.01
	ko00360 Phenylalanine metabolism	3.06E-03	3.24E-03	3.07E-03	-	-	-	<0.01	0.27	0.17	0.42	<0.01	<0.01
	ko00380 Tryptophan metabolism	2.17E-03	2.20E-03	2.05E-03	-	-	-	<0.01	1.49	0.05	0.42	<0.01	<0.01
	ko00400 Phenylalanine, tyrosine and tryptophan biosynthesis	5.57E-03	5.82E-03	5.80E-03	2.1±0.08	2.13±0.05	2.27±0.05	<0.01	<0.01	0.41	0.25	0.05	<0.01
1.6 Metabolism of other amino acids	ko00410 beta-Alanine metabolism	1.97E-03	2.04E-03	2.00E-03	-	-	-	<0.01	0.2	0.05	0.21	<0.01	<0.01
	ko00430 Taurine and hypotaurine metabolism	1.04E-03	1.02E-03	9.60E-04	-	-	-	0.06	1.37	0.05	0.28	<0.01	<0.01
	ko00440 Phosphonate and phosphinate metabolism	3.97E-04	4.27E-04	3.59E-04	-	-	-	<0.01	0.45	0.05	0.3	<0.01	<0.01
	ko00450 Selenocompound metabolism	4.54E-03	4.53E-03	4.56E-03	4.15±0.17	4.05±0.23	4.07±0.05	<0.01	0.12	0.05	0.12	<0.01	<0.01
	ko00460 Cyanoamino acid metabolism	2.11E-03	2.06E-03	2.10E-03	1.38±0.03	1.44±0.03	1.42±0.03	0.07	0.27	0.05	0.12	<0.01	<0.01
	ko00471 D-Glutamine and D-glutamate metabolism	1.13E-03	1.10E-03	1.10E-03	-	-	-	-	-	-	-	-	-
	ko00472 D-Arginine and D-ornithine metabolism	1.14E-05	7.46E-06	1.39E-05	-	-	-	-	-	-	-	-	-
	ko00473 D-Alanine metabolism	1.06E-03	1.06E-03	1.03E-03	-	-	-	-	-	-	-	-	-
	ko00480 Glutathione metabolism	1.80E-03	1.77E-03	1.80E-03	-	-	-	0.1	0.21	0.05	0.12	<0.01	<0.01
1.7 Glycan biosynthesis and metabolism	ko00510 N-Glycan biosynthesis	4.78E-04	4.50E-04	4.92E-04	-	-	-	-	-	-	-	-	-
	ko00511 Other glycan degradation	1.92E-03	2.02E-03	2.16E-03	1.67±0.06	1.78±0.13	1.84±0.12	<0.01	<0.01	0.06	0.09	0.05	<0.01
	ko00513 Various types of N-glycan biosynthesis	1.68E-04	2.53E-04	1.96E-04	-	-	-	-	-	-	-	-	-
	ko00531 Glycosaminoglycan degradation	6.99E-04	7.70E-04	8.13E-04	-	-	-	-	-	-	-	-	-
	ko00540 Lipopolysaccharide biosynthesis	4.68E-04	3.69E-04	4.30E-04	-	-	-	0.63	0.21	0.04	0.07	<0.01	<0.01
	ko00550 Peptidoglycan biosynthesis	8.49E-03	8.37E-03	8.43E-03	3.45±0.39	3.81±0.03	3.52±0.17	0.15	0.26	0.04	0.12	<0.01	<0.01
	ko00563 Glycosylphosphatidylinositol (GPI)-anchor biosynthesis	6.42E-11	0.00E+00	0.00E+00	-	-	-	-	-	-	-	-	-
	ko00603 Glycosphingolipid biosynthesis - globo and isoglobo series	8.25E-04	9.15E-04	9.23E-04	-	-	-	-	-	-	-	-	-
	ko00604 Glycosphingolipid biosynthesis - ganglio series	1.64E-04	2.49E-04	1.90E-04	-	-	-	-	-	-	-	-	-
1.8 Metabolism of cofactors and vitamins	ko00130 Ubiquinone and other terpenoid-quinone biosynthesis	6.25E-04	6.45E-04	5.63E-04	-	-	-	<0.01	0.49	0.05	0.25	<0.01	<0.01
	ko00670 One carbon pool by folate	3.39E-03	3.47E-03	3.39E-03	2.98±0.08	3.13±0.06	2.92±0.02	<0.01	0.27	0.11	0.42	<0.01	<0.01
	ko00730 Thiamine metabolism	3.74E-03	3.76E-03	3.85E-03	2.75±0.03	2.88±0.08	2.9±0.05	<0.01	<0.01	0.05	0.07	<0.01	<0.01
	ko00740 Riboflavin metabolism	1.11E-03	1.17E-03	1.18E-03	-	-	-	<0.01	<0.01	0.06	0.12	<0.01	<0.01
	ko00750 Vitamin B6 metabolism	1.13E-03	1.14E-03	1.10E-03	-	-	-	<0.01	0.45	0.05	0.25	<0.01	<0.01
	ko00760 Nicotinate and nicotinamide metabolism	3.29E-03	3.19E-03	3.17E-03	1.62±0.03	1.44±0.03	1.46±0.05	0.63	1.28	0.04	0.12	<0.01	<0.01
	ko00770 Pantothenate and CoA biosynthesis	3.51E-03	3.54E-03	3.56E-03	3.06±0.08	3.09±0.01	2.95±0.03	<0.01	0.04	0.05	0.12	<0.01	<0.01
	ko00780 Biotin metabolism	2.46E-03	2.53E-03	2.49E-03	1.83±0.08	1.87±0.03	2±0.04	<0.01	0.04	0.09	0.25	<0.01	<0.01
	ko00785 Lipoic acid metabolism	1.04E-04	1.09E-04	8.18E-05	-	-	-	-	-	-	-	-	-
	ko00790 Folate biosynthesis	2.39E-03	2.33E-03	2.37E-03	-	-	-	0.21	0.21	0.04	0.11	<0.01	<0.01
	ko00830 Retinol metabolism	5.78E-04	5.79E-04	5.62E-04	-	-	-	-	-	-	-	-	-
	ko00860 Porphyrin and chlorophyll metabolism	5.75E-03	5.40E-03	6.35E-03	-	-	-	3.91	<0.01	<0.01	<0.01	3.13	10.7
1.9 Metabolism of terpenoids and polyketides	ko00253 Tetracycline biosynthesis	2.37E-06	1.96E-06	1.16E-06	-	-	-	-	-	-	-	-	-
	ko00281 Geraniol degradation	4.34E-04	4.62E-04	4.25E-04	-	-	-	<0.01	0.27	0.05	0.25	<0.01	<0.01
	ko00522 Biosynthesis of 12-, 14- and 16-membered macrolides	1.38E-05	1.59E-05	1.45E-05	-	-	-	-	-	-	-	-	-
	ko00523 Polyketide sugar unit biosynthesis	1.08E-03	1.06E-03	1.06E-03	-	-	-	-	-	-	-	-	-
	ko00900 Terpenoid backbone biosynthesis	3.35E-03	3.29E-03	3.31E-03	1.65±0.12	2.18±0.04	1.66±0.15	0.07	0.27	0.05	0.12	<0.01	<0.01
	ko00902 Monoterpenoid biosynthesis	9.75E-08	2.60E-08	2.53E-07	-	-	-	-	-	-	-	-	-
	ko00903 Limonene and pinene degradation	4.80E-04	4.51E-04	4.47E-04	-	-	-	-	-	-	-	-	-
	ko00906 Carotenoid biosynthesis	8.33E-05	7.24E-05	5.51E-05	-	-	-	0.03	0.27	0.05	0.12	<0.01	<0.01
	ko00908 Zeatin biosynthesis	2.73E-04	2.65E-04	2.67E-04	-	-	-	-	-	-	-	-	-
	ko00909 Sesquiterpenoid and triterpenoid biosynthesis	1.20E-05	1.81E-05	1.16E-05	-	-	-	-	-	-	-	-	-
	ko00981 Insect hormone biosynthesis	2.95E-04	2.48E-04	2.72E-04	-	-	-	-	-	-	-	-	-
	ko01051 Biosynthesis of ansamycins	7.63E-04	7.17E-04	7.48E-04	-	-	-	0.38	0.25	0.04	0.08	<0.01	<0.01
	ko01052 Type I polyketide structures	6.53E-05	6.90E-05	7.56E-05	-	-	-	<0.01	0.09	0.05	0.11	<0.01	<0.01
	ko01053 Biosynthesis of siderophore group nonribosomal peptides	1.18E-03	1.11E-03	1.13E-03	-	-	-	0.35	0.45	0.04	0.11	<0.01	<0.01

	ko01054 Nonribosomal peptide structures	1.09E-03	8.95E-04	1.44E-03	1.53±0.15	1.3±0.15	2.1±0.22	2.55	<0.01	<0.01	<0.01	1.24	6.98
	ko01055 Biosynthesis of vancomycin group antibiotics	4.51E-04	4.55E-04	4.55E-04	-	-	-	-	-	-	-	-	-
	ko01056 Biosynthesis of type II polyketide backbone	2.36E-06	1.94E-06	1.15E-06	-	-	-	-	-	-	-	-	-
	ko01057 Biosynthesis of type II polyketide products	1.83E-05	1.66E-05	2.04E-05	-	-	-	-	-	-	-	-	-
	ko01059 Biosynthesis of enediyne antibiotics	2.56E-05	2.19E-05	2.18E-05	-	-	-	-	-	-	-	-	-
	ko00232 Caffeine metabolism	5.89E-06	2.71E-06	2.61E-06	-	-	-	-	-	-	-	-	-
	ko00261 Monobactam biosynthesis	1.75E-03	1.70E-03	1.78E-03	-	-	-	0.58	0.04	0.04	0.03	<0.01	<0.01
	ko00311 Penicillin and cephalosporin biosynthesis	8.53E-05	7.58E-05	8.07E-05	-	-	-	-	-	-	-	-	-
	ko00331 Clavulanic acid biosynthesis	5.69E-11	0.00E+00	0.00E+00	-	-	-	-	-	-	-	-	-
	ko00332 Carbapenem biosynthesis	5.56E-04	5.54E-04	5.39E-04	-	-	-	-	-	-	-	-	-
	ko00401 Novobiocin biosynthesis	1.56E-03	1.66E-03	1.61E-03	-	-	-	-	-	-	-	-	-
	ko00404 Staurosporine biosynthesis	4.85E-07	3.62E-08	1.15E-07	-	-	-	-	-	-	-	-	-
	ko00405 Phenazine biosynthesis	4.15E-04	4.44E-04	4.54E-04	-	-	-	-	-	-	-	-	-
	ko00521 Streptomycin biosynthesis	2.33E-03	2.25E-03	2.30E-03	2.07±0.06	2.08±0.05	2.19±0.02	0.98	0.45	0.02	0.08	<0.01	<0.01
	ko00524 Neomycin, kanamycin and gentamicin biosynthesis	4.70E-04	4.62E-04	4.73E-04	-	-	-	-	-	-	-	-	-
**1.10 Biosynthesis of other secondary metabolites**	ko00525 Acarbose and validamycin biosynthesis	7.47E-04	7.38E-04	7.45E-04	-	-	-	-	-	-	-	-	-
	ko00901 Indole alkaloid biosynthesis	6.83E-07	1.33E-08	1.04E-07	-	-	-	-	-	-	-	-	-
	ko00940 Phenylpropanoid biosynthesis	1.34E-03	1.25E-03	1.32E-03	-	-	-	-	-	-	-	-	-
	ko00941 Flavonoid biosynthesis	7.34E-06	7.37E-06	8.75E-06	-	-	-	-	-	-	-	-	-
	ko00943 Isoflavonoid biosynthesis	1.95E-07	2.35E-07	1.09E-07	-	-	-	-	-	-	-	-	-
	ko00944 Flavone and flavonol biosynthesis	1.19E-04	1.12E-04	1.31E-04	-	-	-	-	-	-	-	-	-
	ko00945 Stilbenoid, diarylheptanoid and gingerol biosynthesis	7.34E-06	7.37E-06	8.75E-06	-	-	-	-	-	-	-	-	-
	ko00950 Isoquinoline alkaloid biosynthesis	9.24E-04	9.84E-04	9.62E-04	-	-	-	-	-	-	-	-	-
	ko00960 Tropane, piperidine and pyridine alkaloid biosynthesis	1.78E-03	1.87E-03	1.87E-03	-	-	-	<0.01	<0.01	0.12	0.12	<0.01	<0.01
	ko00965 Betalain biosynthesis	8.37E-07	1.41E-07	1.90E-07	-	-	-	-	-	-	-	-	-
	ko00966 Glucosinolate biosynthesis	3.31E-04	3.33E-04	3.38E-04	-	-	-	-	-	-	-	-	-
	ko00361 Chlorocyclohexane and chlorobenzene degradation	4.01E-05	2.91E-05	3.53E-05	-	-	-	0.06	0.21	0.05	0.12	<0.01	<0.01
	ko00362 Benzoate degradation	1.64E-03	1.66E-03	1.66E-03	-	-	-	<0.01	0.21	0.05	0.12	<0.01	<0.01
	ko00363 Bisphenol degradation	4.42E-07	7.79E-07	3.60E-07	-	-	-	-	-	-	-	-	-
	ko00364 Fluorobenzoate degradation	1.89E-05	1.10E-05	1.46E-05	-	-	-	0.06	0.21	0.05	0.12	<0.01	<0.01
	ko00621 Dioxin degradation	1.03E-04	9.41E-05	1.11E-04	-	-	-	0.06	0.09	0.05	0.09	<0.01	<0.01
	ko00622 Xylene degradation	1.53E-04	1.42E-04	1.57E-04	-	-	-	0.06	0.14	0.05	0.11	<0.01	<0.01
	ko00623 Toluene degradation	5.80E-05	4.82E-05	5.25E-05	-	-	-	0.03	0.21	0.05	0.12	<0.01	<0.01
	ko00624 Polycyclic aromatic hydrocarbon degradation	2.99E-05	1.84E-05	2.19E-05	-	-	-	0.06	0.21	0.05	0.12	<0.01	<0.01
	ko00625 Chloroalkane and chloroalkene degradation	2.63E-03	2.59E-03	2.58E-03	1.32±0.07	1.27±0.1	1.12±0.05	0.28	0.57	0.04	0.12	<0.01	<0.01
**1.11 Xenobiotics biodegradation and metabolism**	ko00626 Naphthalene degradation	1.95E-03	1.92E-03	1.92E-03	-	-	-	0.28	0.45	0.04	0.12	<0.01	<0.01
	ko00627 Aminobenzoate degradation	5.83E-04	6.10E-04	5.26E-04	-	-	-	<0.01	0.46	0.05	0.25	<0.01	<0.01
	ko00633 Nitrotoluene degradation	9.29E-04	1.07E-03	9.74E-04	-	-	-	<0.01	<0.01	0.3	0.36	<0.01	<0.01
	ko00642 Ethylbenzene degradation	1.44E-04	1.38E-04	1.54E-04	-	-	-	-	-	-	-	-	-
	ko00643 Styrene degradation	3.48E-04	3.77E-04	3.41E-04	-	-	-	<0.01	0.27	0.05	0.25	<0.01	<0.01
	ko00791 Atrazine degradation	4.32E-04	4.61E-04	4.09E-04	-	-	-	<0.01	0.55	0.05	0.34	<0.01	<0.01
	ko00930 Caprolactam degradation	3.59E-04	3.85E-04	3.29E-04	-	-	-	<0.01	0.49	0.05	0.26	<0.01	<0.01
	ko00980 Metabolism of xenobiotics by cytochrome P450	6.00E-04	5.86E-04	5.72E-04	-	-	-	-	-	-	-	-	-
	ko00982 Drug metabolism - cytochrome P450	6.70E-04	6.50E-04	6.40E-04	-	-	-	-	-	-	-	-	-
	ko00983 Drug metabolism - other enzymes	2.29E-03	2.22E-03	2.29E-03	-	-	-	0.51	0.21	0.04	0.08	<0.01	<0.01
	ko00984 Steroid degradation	4.76E-05	4.43E-05	2.45E-05	-	-	-	<0.01	0.27	0.05	0.12	<0.01	<0.01
